# Study of the antimalarial activity of 4-aminoquinoline compounds against chloroquine-sensitive and chloroquine-resistant parasite strains

**DOI:** 10.1007/s00894-018-3755-z

**Published:** 2018-08-17

**Authors:** Alexandre S. Lawrenson, David L. Cooper, Paul M. O’Neill, Neil G. Berry

**Affiliations:** Department of Chemistry, University of Liverpool, Liverpool, L69 7ZD UK

**Keywords:** Malaria, Antimalarials, QSAR, K1, NF54, Aminoquinolines

## Abstract

This study is concerned with identifying features of 4-aminoquinoline scaffolds that can help pinpoint characteristics that enhance activity against chloroquine-resistant parasites. Statistically valid predictive models are reported for a series of 4-aminoquinoline analogues that are active against chloroquine-sensitive (NF54) and chloroquine-resistant (K1) strains of *Plasmodium falciparum*. Quantitative structure activity relationship techniques, based on statistical and machine learning methods such as multiple linear regression and partial least squares, were used with a novel pruning method for the selection of descriptors to develop robust models for both strains. Inspection of the dominant descriptors supports the hypothesis that chemical features that enable accumulation in the food vacuole of the parasite are key determinants of activity against both strains. The hydrophilic properties of the compounds were found to be crucial in predicting activity against the chloroquine-sensitive NF54 parasite strain, but not in the case of the chloroquine-resistant K1 strain, in line with previous studies. Additionally, the models suggest that ‘softer’ compounds tend to have improved activity for both strains than do ‘harder’ ones. The internally and externally validated models reported here should also prove useful in the future screening of potential antimalarial compounds for targeting chloroquine-resistant strains.

**Electronic supplementary material:**

The online version of this article (10.1007/s00894-018-3755-z) contains supplementary material, which is available to authorized users.

**Graphical Abstract:**
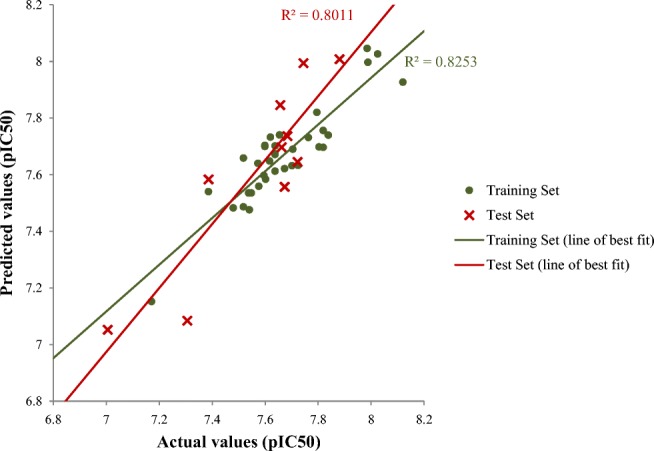
Predictive models reveal linear relationships for activity of 4-aminoquinoline analogues active against chloroquine-sensitive strains of *Plasmodium falciparum*

## Introduction

Malaria is a life-threatening disease, which, according to the World Health Organization (WHO), resulted in almost half a million deaths in 2016, with 91% of those occurring in the WHO African Region [1]. Malaria is spread through the bite of the female *Anopheles* mosquito, with symptoms only becoming apparent several days later. Of the five *Plasmodium* parasites known to cause malaria (*P. falciparum*, *P. vivax*, *P. malariae*, *P. ovale*, *P. knowlesi*), *P. falciparum* is the most deadly form, which, if not treated, can lead to severe illness and possible death. Upon taking a blood meal, the mosquito injects into the human host sporozoites that invade the liver cells and undergo development and multiplication [2]. The eventual rupture of the hepatocytes releases merozoites into the blood, which go on to enter the red blood cells and undergo further maturation and multiplication. The rupture of these blood cells results in the characteristic cyclical fever associated with malaria. During maturation in the red blood cells, the parasite remodels the host cell by inserting parasite proteins and phospholipids into the red blood cell membranes [3]. The host hemoglobin is also digested and transported to the parasite food vacuole, where it provides a source of amino acids. Free heme is generated during this process and is ordinarily toxic to the parasite, but it is readily converted to hematin and subsequently undergoes dimerization to form β-hematin. The majority of this β-hematin is then rendered harmless to the parasite through biocrystallization to form insoluble hemozoin [4].

**Fig. 1:**
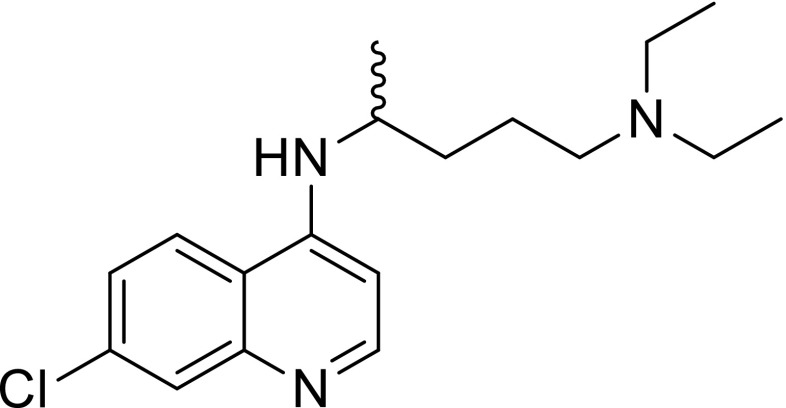
Chloroquine

The 4-aminoquinoline drug chloroquine (Fig. 1) has found widespread use in the treatment of malaria [5]. Chloroquine is thought to become trapped within the parasite food vacuole, where it prevents the biocrystallization of β-hematin. This occurs due to the acidic nature of the vacuole, in which chloroquine becomes ‘trapped’ in its membrane-impermeable doubly protonated form. Chloroquine then forms a complex with free heme, leading to the accumulation of heme and, ultimately, parasite death. Unfortunately, *Plasmodium falciparum* is now resistant to chloroquine in most parts of the world [6, 7]. In resistant strains of the parasite, chloroquine can escape from the parasite food vacuole due to a mutation in the *Pf*crt gene that encodes a protein known as the chloroquine resistance transporter (*Pf*crt) [8, 9]. This transporter protein causes decreased accumulation of the drug within the food vacuole due to alterations in the membrane protein, which allow chloroquine to diffuse away from the vacuole. Chloroquine-resistant strains of the parasite possess a neutral threonine residue in place of the positively charged lysine moiety at position 76 of the *Pf*crt protein, thereby allowing the chloroquine to exit the food vacuole down a steep outward concentration gradient [10–12]. Structural modifications to the 4-aminoquinoline chemotype can produce analogues that circumvent chloroquine resistance and which exhibit similar antimalarial potential [13].

As is well known, quantitative structure–activity relationship (QSAR) models can be used to correlate the structural and physicochemical features of a molecule with a measured property of interest such as biological activity [14]. The foundations of QSAR rest upon the similarity principle, which suggests that structurally similar compounds are more likely to exhibit similar properties [15]. The molecular descriptors used in QSAR studies describe the various chemical and physical properties of the compounds which, when expressed numerically, can form quantitative relationships with activity. When a relationship is found, the resulting mathematical expression can predict the biological activity of other chemical structures that were not used to develop the model, and whose biological activity is as yet unknown. Katritzky et al. [16] successfully used QSAR techniques to model the antimalarial activity of two diverse sets of compounds against different parasite strains, with the descriptors utilized in their models being related to the mechanism of action of the compounds.

The research presented here details the development of QSAR models for a series of compounds that contain the 4-aminoquinoline motif and which have previously been tested against both a chloroquine-sensitive (NF54) and chloroquine-resistant (K1) strain of malaria. Our aim was to develop robust QSAR models that are capable of predicting activities for both of these strains. A range of machine learning methods was used, alongside rigorous validation of the resulting models. Subsequent interpretation of the molecular descriptors in terms of the mode of action of the 4-aminoquinoline compounds provides useful guidance as to how to circumvent parasite resistance to this class of compound.

## Methods

### The 4-aminoquinoline dataset

**Fig. 2:**
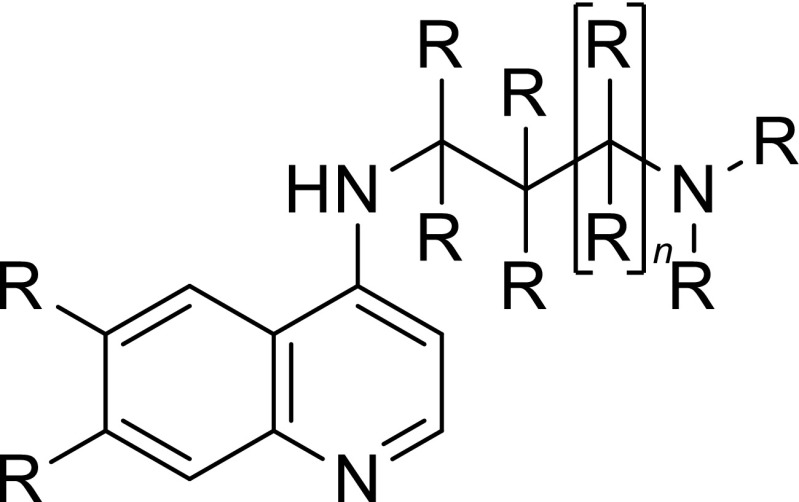
The 4-aminoquinoline template, with R groups ranging from simple H or Cl atoms to alkyl substitutions and trifluoromethyl groups, and *n* either 0 or 1

The structures and biological testing results of chloroquine and of 44 novel 4-aminoquinoline compounds of the general formula shown in Fig. 2 are described in US patent 5596002 [17]. The selected *P. falciparum* strains included the MDR chloroquine-resistant strain K1 and the sensitive strain NF54, which were selected on the basis of genotypic and phenotypic information previously reported in the literature [18]. Quantitative IC_50_ values have been reported for all 45 compounds against the NF54 and K1 parasite strains, with activities ranging from 2 ng/ml to 30 ng/ml for the NF54 strain and from 6 ng/ml to 114 ng/ml for K1 [17]. The chemical structures, alongside these activity data, are displayed in Table S1 (Supporting Information). After conversion of these IC_50_ values to mol dm^−3^, we generated pIC_50_ = −lg(IC_50_) values for each of the 45 compounds against both parasite strains.

The Spartan′08 package [19] was used with the MMFF94 molecular mechanics force field [20] to generate an energy minimum conformation for each of the 45 compounds. Molecular descriptors describing the 0, 1 and 2 dimensional properties of the compounds, encompassing information such as constitutional counts, chemical functionality, and topological features, were then calculated using both DRAGON [21] and ADMEWORKS Modelbuilder [22]. This resulted in a set of 957 descriptors.

**Fig. 3:**
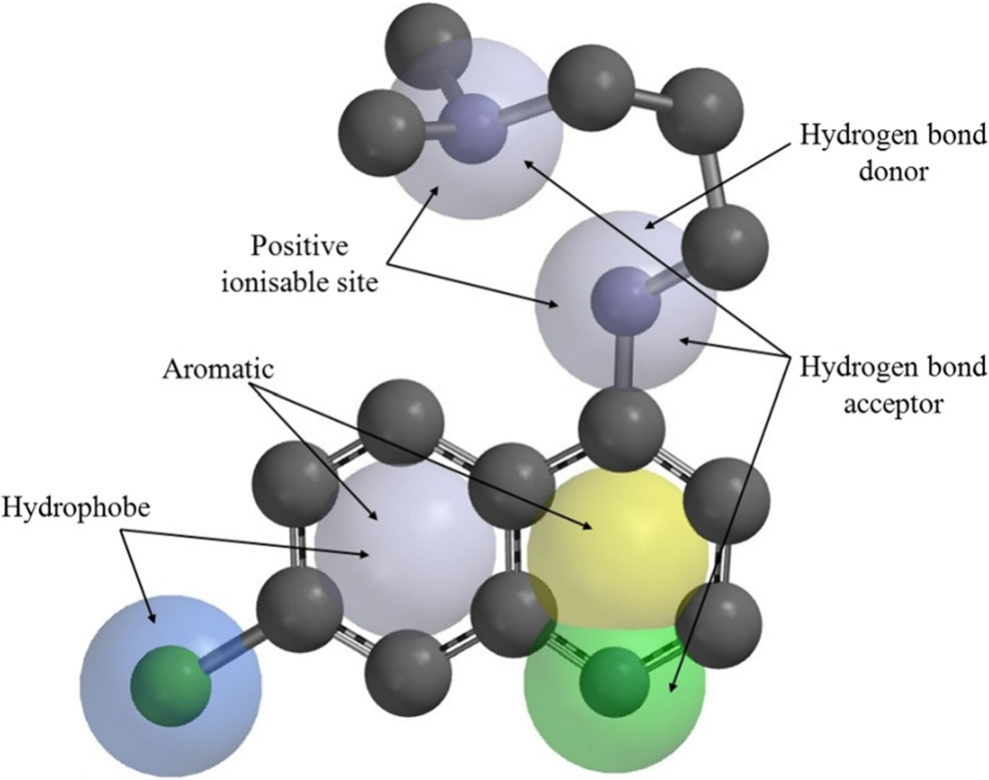
Pharmacophore shown with chemical functions labels (H atoms removed for clarity)

Spartan′08 [19] was used to perform a conformational search and subsequent structural alignment of the 4-aminoquinoline compounds to a common pharmacophore. This pharmacophore was defined based on the lowest energy conformation of the most active compound across the two strains (molecule 15 in Table S1), using chemical functional descriptors as shown in Fig. 3. Following the generation of a conformer library, a similarity analysis was performed for each of the other 44 compounds so as to identify the conformation closest to that of the pharmacophore. An additional 673 three-dimensional (3D) molecular descriptors, which encode for important features such as structural geometry and molecular surfaces, were calculated for the resulting structures.

For each strain, the resulting datasets of 0 to 3D descriptors (1630 in total) were auto-scaled: each descriptor is divided by the standard deviation for that descriptor across all observations, such that each scaled descriptor then has a mean of 0 and a variance of 1 [14]. Such normalization of the variables allows the (scaled) descriptors to be compared on an equal footing. Although it has been shown that the exclusion of 3D molecular descriptors can still yield significant QSARs [23], this seems unlikely to be the case for the present study. This is because several of the molecules considered here (see Table S1) contain a stereogenic carbon atom, with different enantiomers displaying different activity values. Nonetheless, we did also seek models based only on the 0 to 2D descriptors (957 in total).

### QSAR generation

Defining appropriate training and test sets is an essential part of the QSAR development process: it allows for models to be built on a training set and then for their performance to be assessed on a test set. Such training and test sets should satisfy various criteria [24], including: (1) representative points of the test set must be close to representative points of the training set; (2) representative points of the training set must be close to those of the test set; and (3) the training set must be diverse. The sphere-exclusion algorithm described by Hudson et al. [25] attempts to meet such criteria, identifying which compounds most effectively cover the available property space. Briefly, the most active compound is selected for the training set, following which all compounds that are within the similarity threshold to the selected compound are placed instead in the test set. This is analogous to removing from the training set all compounds that are enclosed in a notional hypersphere centered on the most active compound. Out of the remaining compounds, the algorithm then identifies the one that lies closest to the center of the hypersphere and places it in the training set. Again, compounds within a similarity threshold are placed instead in the test set. This process continues until there are no more compounds to select.

Appropriate feature selection, using both objective and subjective methods, was applied to the datasets of molecular descriptors. In general terms, objective selection techniques aim to remove molecular descriptors which are irrelevant or redundant, so as to minimize multicollinearity. A key benefit is a lower probability of chance correlations, which are possible when there are more descriptors than data points. The selection strategy involves removing those descriptors that are highly correlated to one another, keeping only the descriptors that provide unique information. The CORCHOP [26] routine was used as the objective method providing a means of systematically reducing the initially large number of descriptors whilst retaining the vital information. So as to reduce the number of molecular descriptors still further, a variety of subjective methods were used to generate subsets [27]. Whilst objective methods simply consider the relationships between the independent variables (descriptors), subjective methods select the most appropriate descriptors based on their relationship to the dependent variable, in this case biological activity, as quantified by the pIC_50_ values. The methods we employed included forward selection, which selects the descriptors which contribute the most to a model in an iterative process, based on their correlation to activity [28]. Backwards elimination was also studied; it involves periodically removing the least informative descriptors until a desired number is reached [14]. Additionally, a stepwise procedure was considered, which is similar to forward selection, except that at each stage the possibility of deleting a descriptor is considered [29]. Finally, a genetic algorithm (GA) was considered that generated a population of linear regression equations, with each having a different combination of descriptors, so that ultimately the best selection can be chosen [30].

We made extensive use of the software package PHAKISO [31] for the generation of QSAR models as well as for data pruning and for the splitting of the data into training and test sets. Default settings were employed except for subjective selection where the maximum/minimum variables was altered so that the molecule to descriptor ratio ≥ 5 and the error measurement was adjusted coefficient of determination. For the various descriptor subsets that were selected, QSAR models were generated using linear methods, starting with multiple linear regression (MLR). Subsequent to MLR generation using PHAKISO [31], programs and scripts were written that can generate and process the MLR equations for all possible combinations of up to 23 descriptors (with the actual MLR statistics obtained using calls to NAG library routines [32]).

Partial least squares (PLS) was examined as an alternative to MLR, given that it can be particularly useful when the number of independent variables is comparable to, or much greater than, the number of data points. The chosen descriptors explain not only the variance in the descriptors, but also in the dependent variable, and can lead to highly stable and predictive models even when there is a high degree of correlation between descriptors [33].

### Model validation

The validation of QSAR models was performed according to standard criteria that are specified throughout the literature. For the internal validation of our models, we required [14, 34–37]: a molecule to descriptor ratio ≥ 5; *r*^2^ ≥ 0.7; ${r}_{LOO}^2$ ≥ 0.5; (*r*^2^ − ${r}_{\mathrm{bootstrap}}^2$) ≤ 0.3; F-statistic > tabulated value; *t*-statistic for each descriptor ≥2. Here, *r*^2^ is the coefficient of determination (squared correlation coefficient) between the predicted and observed pIC_50_ values; ${r}_{LOO}^2$ is the leave-one-out (LOO) cross-validation value of *r*^2^, found when a single data point is removed and a new model is calculated, with the new model being used to predict the dependent variable of the removed object; ${r}_{\mathrm{bootstrap}}^2$ is determined by resampling the initial data and generating new models, which can then predict the excluded samples, with a high ${r}_{\mathrm{bootstrap}}^2$ value generally being indicative of a robust model [38].

Internal validation allows an assessment of the robustness of a model, but gives no true measure as to its predictive capabilities. Instead, external validation on a test set not used during the model development is realistically the only truly predictive test [38]. Accordingly, the models were applied in the present work to the test sets that were generated during the splitting of the original data. The following standard criteria were applied: *q*^2^ > 0.5; *r*^2^ > 0.6; |*r*_0_^2^ – *r*_0_′^2^| < 0.3; (*r*^2^-*r*_0_^2^)/*r*^2^ < 0.1; 0.85 ≤ *k* ≤ 1.15 or 0.85 ≤ *k*′ ≤ 1.15. Here, *q*^2^ represents the LOO cross-validated *r*^2^ for the test set. The coefficient of determination and the gradient of the best-fit line passing through the origin are denoted *r*_0_^2^ and *k*, respectively, for the predicted against observed pIC_50_ values. The primed quantities, *r*_0_′^2^ and *k*′, signify the analogous quantities for the observed against predicted values. The family of QSAR models developed after different random shuffling of the dependent variable (Y-randomization) should of course generally have inferior training statistics [38]. Such Y-randomization tests were performed for our more promising models. The relative weights of the descriptors within significant regression models were calculated by closely approximating the average increase in *r*^2^ obtained by adding a predictor variable across all possible sub-models [39]. This allows for the proportion of the variance accounted for by the model to be divided amongst the independent variables.

## Results and discussion

### NF54 strain

Various encouraging models could be generated using the 0 to 3D descriptors (Table 1, models 1–4), with GA-MLR identifying the most promising QSARs. Applying the principle of parsimony, we judged that model 4 was better than model 3, with all descriptors possessing a *t*-statistic greater than 2.

**Table 1:** Summary of key statistics for the NF54 and K1 MLR models. Results given to two decimal places

Model	Descriptor set	Method	# descs^a^	Training set statistics					Test set statistics			
				*r* ^2^	*q* ^2^	F-statistic	${r}_{\mathrm{bootstrap}}^2$	*q* ^2^	*r* ^2^	(*r*^2^–*r*_0_^2^)/*r*^2^	*k*	|*r*^2^–*r*_0_^2^|
NF54:												
1	0-3D	Forward	12	0.72	0.41	5.76	−0.80	0.02	0.62	0.02	0.99	0.48
2	0-3D	Stepwise	3	0.60	0.31	18.27	−1.20	0.86	0.97	0.04	1.00	0.05
3	0-3D	GA-MLR	8	0.84	0.74	20.47	0.66	0.56	0.72	0.00	1.00	0.14
4	0-3D	GA-MLR	7	0.75	0.57	13.48	0.43	0.60	0.73	0.00	1.00	0.09
5	(38)^a^	GA-MLR	10	0.88	−1.04	17.73	0.91	0.63	0.74	0.00	1.01	0.12
6	(38)	GA-MLR	9	0.89	0.31	22.75	0.89	0.62	0.74	0.00	1.01	0.04
7	(38)	GA-MLR	8	0.84	−4.57	17.28	0.87	0.67	0.73	0.00	1.00	0.08
8	(38)	GA-MLR	7	0.83	−4.70	19.30	0.87	0.64	0.79	0.00	1.01	0.07
9	(38)	GA-MLR	6	0.78	−2.20	16.89	0.81	0.63	0.74	0.00	1.00	0.10
10	(38)	GALib-MLR	10	0.90	0.69	20.70	0.59	0.75	0.88	0.03	1.00	0.11
11	(38)	GALib-MLR	7	0.83	0.53	18.23	0.70	0.68	0.80	0.01	1.01	0.12
12	(38)	GALib-MLR	8	0.85	0.59	18.54	0.61	0.64	0.86	0.05	1.01	0.17
K1:												
13	0-3D	GA-MLR	9	0.89	0.82	25.67	0.72	0.20	0.31	1.04	1.00	0.21
14	(33)^a^	GA-MLR	8	0.91	0.83	35.09	0.78	0.72	0.75	0.02	1.00	0.01
15	(33)	GA-MLR	7	0.91	0.83	33.48	0.72	0.57	0.68	0.00	1.00	0.11
16	(33)	Stepwise	3	0.68	0.56	22.45	0.53	0.89	0.89	0.04	1.00	0.03
17	(33)	Stepwise	3	0.74	0.62	25.06	0.56	0.59	0.68	0.02	1.00	0.05

^a^(38) and (33) denote models created from the pruned subsets of 38 and 33 descriptors, with # descs being the actual number of descriptors used to build the model

In order to identify the most significant independent variables, a descriptor selection method was adopted in which QSAR models were developed independently using either the DRAGON [21] or ADMEWORKS Modelbuilder [22] descriptor sets (plus 3D descriptors). Using GA-MLR, a series of eight models based on between seven and ten descriptors was developed and validated internally for all 45 compounds (i.e., there were no test sets). Descriptors with a *t*-statistic > 2 were then collated into a new subset of descriptors. It was hoped that more statistically valid models could be found when using the resulting set of 38 descriptors.

**Fig. 4:**
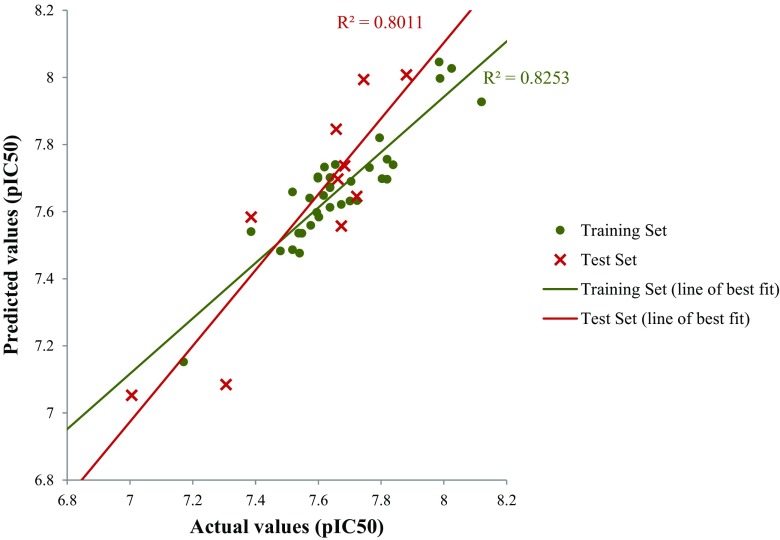
Linear relationships observed in model 11 for NF54 data

Models were initially developed with GA-MLR (Table 1, models 5–9) using these 38 descriptors but they failed to satisfy all validation criteria. On the other hand, an alternative subjective selection method, GALib [40], resulted in three models (Table 1, models 10–12) that passed all of the required validation criteria, with strong statistics observed throughout. Figure 4 illustrates the linear relationship that was observed for model 11, clearly showing the favorable performance for both the training and test set.

**Fig. 5:**
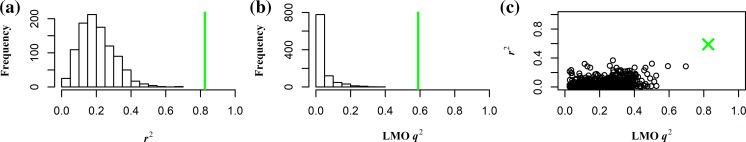
**a–c** Y-randomization and leave-many-out (LMO) *q*^2^ validation (1000 iterations) tests for model 11. **a** *Histogram* showing the effect of Y-randomization on the *r*^2^ statistic, with the better value for the actual model shown by the *green vertical line*. **b** *Histogram* showing the effect of Y-randomization on the LMO *q*^2^ statistic, with the better value for the actual model shown by the *green vertical line*. **c** *Scatter plot* of *r*^2^ values against LMO *q*^2^ values, with the actual model (*green cross*) clearly performing better those generated by Y-randomization

Further validation was sought for models 10–12 using the leave-many-out (LMO) cross validation approach. A mean *q*^2^ was calculated over 1000 iterations, with a value exceeding 0.5 generally considered to be a significant cut-off point for good models [34]. Y-randomization of both the *r*^2^ and LMO *q*^2^ statistics was also performed to check that the models were robust and not simply down to chance correlations. It is clear from the results shown in Table 2 that models 10–12 all pass the thresholds. The plots collected in Fig. 5 demonstrate very clearly for model 11 that the alternative models generated through Y-randomization consistently have much poorer statistics than does the actual model. The corresponding plots for models 10 and 12 have similar characteristics. These three models can therefore be considered robust and statistically significant with regard to predicting the NF54 activity of these 4-aminoquinoline compounds.

**Table 2:** Summary of Y-randomization and leave-many-out (LMO) *q*^2^ validation tests for models 10–12 and 14–17 (using 1000 iterations)

Model	Method	*r* ^2^	Average *r*^2^ (Y-randomization)	LMO *q*^2^	Average LMO *q*^2^ (Y-randomization)
NF54:					
10	GALib-MLR	0.90	0.30 ± 0.12	0.59	0.035 ± 0.053
11	GALib-MLR	0.83	0.21 ± 0.11	0.59	0.034 ± 0.051
12	GALib-MLR	0.85	0.23 ± 0.11	0.52	0.031 ± 0.046
K1:					
14	GA-MLR	0.91	0.23 ± 0.10	0.82	0.044 ± 0.055
15	GA-MLR	0.91	0.23 ± 0.11	0.82	0.051 ± 0.067
16	Stepwise	0.68	0.083 ± 0.067	0.56	0.050 ± 0.063
17	Stepwise	0.74	0.099 ± 0.075	0.62	0.058 ± 0.074

As we had anticipated, QSAR analysis of the NF54 dataset using just 0 to 2D descriptors was unsuccessful. A single model found using GA-MLR displayed some promise, with an internal *r*^2^ value of 0.80, but it failed to meet the acceptance criteria when tested externally, showing no predictive capabilities.

### K1 strain

Much the same strategies were applied to the K1 data, but this time only one internally valid model was found using GA-MLR with the 0 to 3D descriptors (Table 1, model 13), and we could not find any externally valid models. (Unsurprisingly, no statistically valid models could be found using just the 0 to 2D descriptors.) It was thought that the large descriptor space may have hindered descriptor selection using objective and subjective methods, so we tried instead our alternative descriptor selection method that proved successful for the NF54 data.

**Fig. 6:**
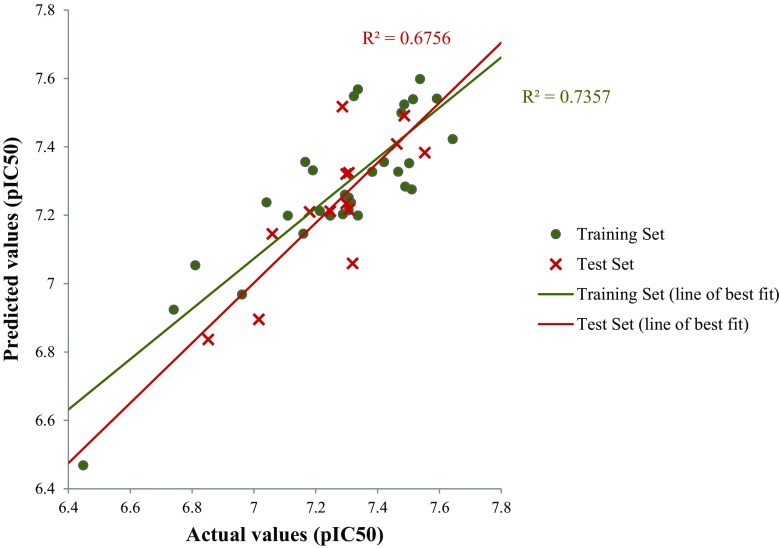
Linear relationships observed in model 17 for K1 data

As before, the descriptors were split into subsets according to which computational program was used to generate them. Models were developed using GA-MLR (with all compounds in the training set) and descriptors with a *t*-statistic > 2 were selected from the eight statistically significant models that we found. This procedure yielded a new ‘pruned’ set of 33 descriptors that could then be used in QSAR development using multiple subjective selection methods. Our approach yielded four internally and externally significant models (Table 1, models 14–17). Just as in the NF54 study, QSAR analysis using the pruned descriptor set produced much more successful models. Figure 6 illustrates the linear relationship that was observed for model 17, showing favorable performance for both the training and test sets.

**Fig. 7:**
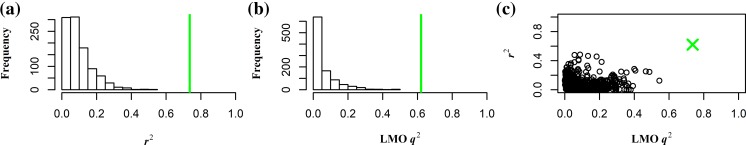
**a–c** Y-randomization and LMO *q*^2^ validation tests (1000 iterations) for model 17. **a** *Histogram* showing the effect of Y-randomization on the *r*^2^ statistic, with the better value for the actual model shown by the *green vertical line*. **b** *Histogram* showing the effect of Y-randomization on the LMO *q*^2^ statistic, with the better value for the actual model shown by the *green vertical line*. **c** *Scatter plot* of *r*^2^ values against LMO *q*^2^ values, with the actual model (*green cross*) clearly performing better those generated by Y-randomization

Although model 16 has an *r*^2^ value of 0.68, slightly below our 0.7 cut-off, we chose to include it because it performs well across all of the other validation criteria. It was validated further, along with the other models, through LMO *q*^2^ and Y-randomization analysis. These results provide further evidence of the predictive nature of the models (Table 2, models 14–17). The plots collected in Fig. 7 demonstrate very clearly for model 17 that the alternative models generated through Y-randomization consistently have much poorer statistics than does the actual model. The corresponding plots for models 14–16 have similar characteristics. All four models (14–17) can thus be considered robust and statistically significant with regard to predicting the K1 activity of these 4-aminoquinoline compounds.

### Descriptor frequency

**Fig. 8:**
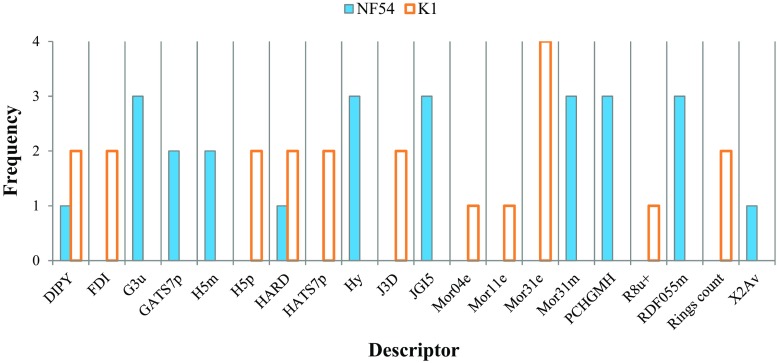
Frequency of descriptor usage in the validated NF54 and K1 models

For each of the descriptors with *t*-statistics > 2 in the validated models of both strains, we determined the total number of times that they were used, with the aim of finding the most commonly occurring descriptors used to predict pIC_50_ values for the two strains. Figure 8 illustrates the results, with some descriptors common to models from both strains, and others unique to one or the other. Six descriptors were present within all three of the NF54 models: G3u, Hy, JGI5, Mor31m, PCHGMH, and RDF055m. Only one descriptor, Mor31e, was present for all four K1 models. Descriptors DIPY and HARD were the only ones common to models for both strains.

To garner further support for the 20 significant descriptors identified by Fig. 8, we examined the MLR models for all possible combinations of these descriptors. From this rather large set of models, further analysis was performed only for those with fewer than ten descriptors (to avoid over-fitting) and that have an *r*^2^(adj) > 0.7 (where the notation *r*^2^(adj) signifies that *r*^2^ has been adjusted in the standard way that takes account of the number of degrees of freedom in the model). The frequency of descriptor usage in the models that met these criteria were found to be concordant with conclusions drawn from Fig. 8. In particular, the descriptor Hy was present across all 41 models for the NF54 strain, with JGI5 present in all but one model. Additionally, the Mor31e descriptor was the only one present across all 61 models for the K1 strain. The HARD descriptor was relatively common for both strains, being present in 25 of the NF54 models and 53 of those for K1.

Analysis of the relative descriptor weights within models 10–12 for NF54, and 14–16 for K1 offered additional support for their importance. Descriptors JGI5 and Hy were consistently found to be those with the greatest relative contribution to *r*^2^ in models 10–12 for NF54, with respective weights of 40.8%, 45.0% and 43.6% for JGI5, and of 13.8%, 21.2% and 19.2% for Hy. Similarly, the Mor31e descriptor, which was present across all regression models for K1, had weights for models 14–17 of 26.5%, 24.6%, 35.0% and 33.9%, respectively. This shows that not only were these the most commonly occurring descriptors across the models, but that they were also the most significant in terms of defining the models.

To assess whether the same descriptors would be found to be as important when using an alternative machine learning method, models were developed using PLS QSAR, for both the NF54 and K1 strains, using the pruned subsets of descriptors (vide supra) of 38 for NF54 and 33 for K1. The best model obtained using PLS is reported in Table 3 for each strain. Both models satisfy the requirement of at least a 5:1 ratio between the number of molecules in the training set and the number of principal components in the model. Information about the relative importance of the descriptors in the principal components (see Table S2 in the Supplementary Information, which also provides brief descriptions of the various descriptors) comes from an examination of the absolute weights. In this way, descriptors Hy and JGI5 were found to be two of the most important descriptors in the NF54 PLS model, with Mor31e being the most important in the corresponding K1 model. Additionally, the HARD descriptor has a strong weight in both models.

**Table 3:** Summary of key statistics for the partial least squares (PLS) models generated using the pruned sets descriptors (38 for NF54 and 33 for K1), with # comps being the number of principal components used in the model. Results mostly given to two decimal places

Model	Strain	# comps	Training set statistics				Test set statistics				
			*r* ^2^	*q* ^2^	F-statistic	${r}_{\mathrm{bootstrap}}^2$	*q* ^2^	*r* ^2^	(*r*^2^–*r*_0_^2^)/*r*^2^	*k*	|*r*^2^–*r*_0_^2^|
18	NF54	7	0.97	0.60	4.04 × 10^9^	0.45	0.72	0.84	0.007	1.01	0.07
19	K1	4	0.98	0.75	5.72 × 10^9^	0.77	0.80	0.85	0.014	1.01	0.01

### Descriptor interpretation

Whether we used MLR or PLS, descriptors Hy and JGI5 were found consistently to be the most influential in describing the activity for NF54, whilst Mor31e was the most influential for K1. The HARD descriptor was common in models for both strains. This identification of the most important molecular descriptors may highlight important differences between the chloroquine-sensitive and chloroquine-resistant strains of the parasite and it could provide useful clues to the resistance mechanism.

**Fig. 9:**
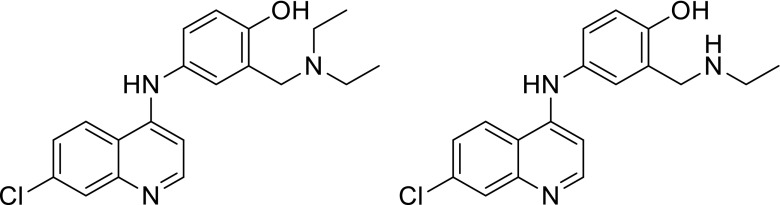
Amodiaquine and desethylamodiaquine

Given that the hydrophobicity of compounds has previously been shown in QSAR analysis to be of importance for influencing antimalarial activity [41], we start by considering the Hy descriptor, which encodes hydrophilicity. This descriptor, which was introduced by Todeschini and Gramatica [42], is a simple empirical index related to the hydrophilicity of the substituents within a compound. We observe that the Hy descriptor features in the NF54 models with a positive coefficient, suggesting that more hydrophilic compounds have improved activity against the NF54 strain, whereas the hydrophilic properties of these 45 compounds appear to have relatively little bearing on their K1 activity. Similarly, it is well established that amodiaquine and its metabolite desethylamodiaquine (see Fig. 9) are equipotent against chloroquine-sensitive parasites [43]. The Hy descriptor values are almost identical for these two compounds. Conversely, desethylamodiaquine is less potent than amodiaquine against chloroquine-resistant strains [43], suggesting that their hydrophilic properties are of lower importance with regard to their antimalarial activity in resistant strains, just as was observed for our K1 models. Another example is provided by hydroxychloroquine, which is a much more hydrophilic analogue of chloroquine with lower log*D* [44] (where log*D* quantifies the distribution of charged states of the compound between organic and aqueous phase at a given pH and thus indicates the degree of lipophilicity). Whereas hydroxychloroquine exhibits similar activity against chloroquine-sensitive strains, it is many times less active than chloroquine against resistant parasites [44].

These various observations are consistent with the known resistance mechanism, namely decreased accumulation of the drug in the food vacuole due to expression of chloroquine resistance transporter [5]. Hydrophilic compounds are likely to be more easily protonated and thus trapped in the food vacuole in the chloroquine-sensitive strain. This trapping is less efficient in the resistant strain, so that the hydrophilicity (and protonation state) of the 4-aminoquinolines is not as important for predicting their K1 activity. The hydrophilicity of these 4-aminoquinolines therefore represents an important consideration to optimize activity against the chloroquine-sensitive NF54 strain, but unfortunately not to overcome chloroquine resistance in the K1 strain.

Clearly there are many important physical, chemical, and biological properties that are related to the charge distribution within a compound. The JGI5 descriptor, which is of significance within the NF54 models, represents the mean Galvez topological charge index of order five [45]. Such topological charge indices were proposed for evaluating the charge transfer between pairs of atoms, and therefore the global charge transfers in a given molecule [46, 47]. Here, the negative coefficient for the JGI5 descriptor in the NF54 models indicates that charge transfer between the atom pairs has a negative influence on activity, so that compounds with lower global charge transfers have improved activity against the NF54 strain.

We found that the most essential descriptor for explaining and predicting the activity of these 4-aminoquinolines molecules against the K1 strain is the Mor31e descriptor, which is defined as a 3D-MoRSE descriptor that encodes for signal 31, weighted by atomic Sanderson electronegativities [45]. Descriptors of this type are based on the idea of obtaining information from the 3D atomic coordinates of a given molecular structure from the transformations used in electron diffraction studies for preparing theoretical scattering curves [48]. As such, it is difficult to interpret the Mor31e descriptor directly in terms of its chemical significance. An additional descriptor that is of moderate frequency across the various MLR and PLS K1 models is the ring count descriptor, with higher values favoring higher K1 activity. This descriptor is found to be in the top two in order of importance in the regression models that contain it, with weights of 22.4% and 29.3%, respectively, in models 14 and 15. It follows from our various observations that the identification of structurally similar 4-aminoquinoline compounds that feature both more rings and low Mor31e descriptor values could prove promising for optimizing activity against K1. The addition of more rings tends to increase log*D*, which has been shown to be a key factor in influencing resistance ratios, with higher log*D* values corresponding to increased activity against K1 [49].

Finally, the HARD descriptor, which was present throughout the successful models for both strains, represents a measure of the hardness of a given compound, i.e., the resistance to change of its electron distribution [50]. It is associated with the hard and soft acids and bases (HSAB) concept, also known as the Pearson acid base concept [51], which is used widely in chemistry for rationalizing the stability of compounds, reaction mechanisms and pathways, and so on. As is well known, the term ‘hard’ is generally used for chemical species that are small, have high charge states and are weakly polarizable, whereas ‘soft’ species tend to be big and strongly polarizable, and to have low charge states. We found that the HARD descriptor enters the NF54 and K1 models with a negative coefficient, so that it is the ‘softer’ molecules that have improved activity against both strains. In general terms, the ‘softer’ molecules tend to be more lipophilic: they have higher log*D* values and so we may expect better activity against K1 [49]. Additionally, ‘softer’ systems may interfere more than do ‘harder’ ones with the β-hematin biocrystallization [52].

## Conclusions

Statistically significant QSAR models have been developed for both the chloroquine-sensitive NF54 and chloroquine-resistant K1 strains using MLR and PLS methods. A novel method for selecting a ‘pruned’ set of optimum descriptors for model development was particularly effective. Several QSAR models were validated statistically and shown to exhibit strong predictive capabilities, so that they may now be used with some confidence to predict the potential activity against both the NF54 and K1 strains of structurally similar 4-aminoquinoline compounds.

Analysis of the frequency of use of the various descriptors within the models proved to be informative, with Hy and JGI5 being used commonly for the NF54 strain, the Mor31e descriptor being present in all K1 models, and the HARD descriptor being common throughout models for both strains. This pattern of descriptor usage can be interpreted in terms of the mode of action of these 4-aminoquinoline compounds, as well as their chloroquine resistance mechanism. In particular, the hydrophilic properties were crucial in predicting NF54 activity, with more hydrophilic compounds (which are likely to be more easily protonated) showing improved potency. This supports the hypothesis that the compounds become trapped in the food vacuole of the malaria parasite, where they elicit their response. Additionally, the descriptor usage suggests that ‘softer’ compounds have higher activity than do ‘harder’ ones. Taken together, these various observations should prove useful in rational drug design, with the direct use of our QSAR models in future virtual screening campaigns aiding the in silico identification of potentially active compounds that merit subsequent synthesis and biological testing.

## Electronic supplementary material

ESM 1 Chemical structures and IC50 values; PLS models for NF54 and K1 pIC50 values. (DOCX 624 kb)
