# Supplementary material for: Study of the antimalarial activity of 4-aminoquinoline compounds against chloroquine-sensitive and chloroquine-resistant parasite strains
Source: J Mol Model. 2018 Aug 17;24(9):237. doi: 10.1007/s00894-018-3755-z (PMC6097041; doi:10.1007/s00894-018-3755-z)
Supplement: Supplementary file 1 — Chemical structures and IC50 values; PLS models for NF54 and K1 pIC50 values. (DOCX 624 kb) [file 894_2018_3755_MOESM1_ESM.docx]

**Supporting Information**

**Study of the antimalarial activity of 4-aminoquinoline compounds against chloroquine sensitive and chloroquine resistant parasite strains**

Alexandre S. Lawrenson, David L. Cooper, Paul M. O’Neill, Neil G. Berry*

Department of Chemistry, University of Liverpool, Liverpool L69 7ZD, UK

*Corresponding author: [ngberry@liverpool.ac.uk](mailto:ngberry@liverpool.ac.uk)

**Contents**

Chemical structures and IC_50_ values 2

Partial least squares models for NF54 and K1 pIC50 values 11

Table S1 The 4-aminoquinoline structures together with their IC_50_ values for the NF54 and K1 strains. RMM denotes the relative molecular mass used in the conversion to pIC50 values (see main text).

| Compound | IC_50_ (ng/ml) | | RMM |
| --- | --- | --- | --- |
|  | NF54 | K1 |  |
| 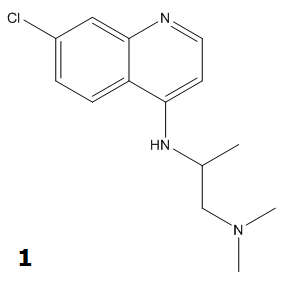 | 4 | 9 | 263.77 |
| 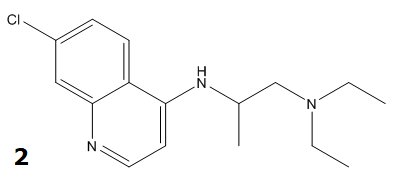 | 7 | 14 | 291.82 |
| 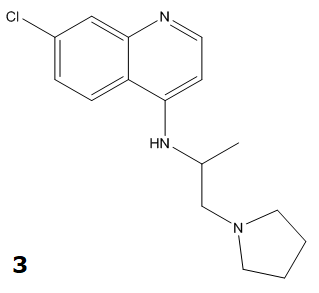 | 7 | 12 | 289.8 |
| 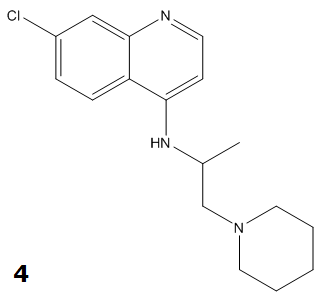 | 7 | 15 | 303.83 |
| 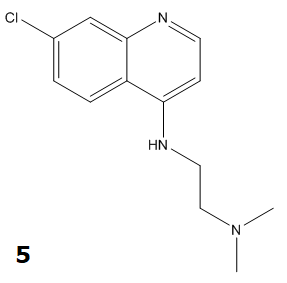 | 4 | 7 | 249.74 |
| 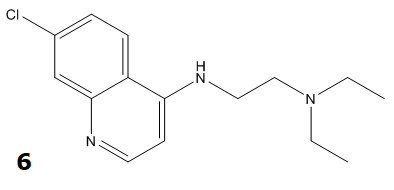 | 4 | 9 | 277.79 |
| 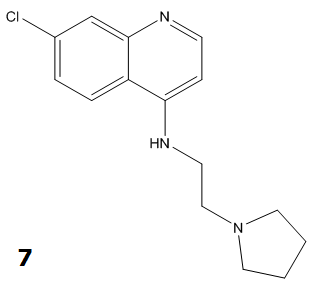 | 4 | 8 | 275.78 |
| 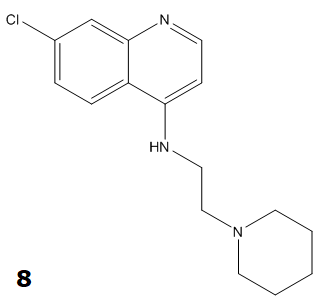 | 5 | 11 | 289.8 |
| 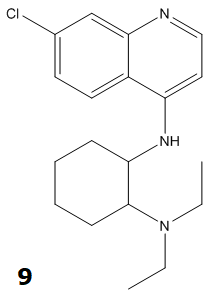 | 11 | 32 | 331.88 |
| 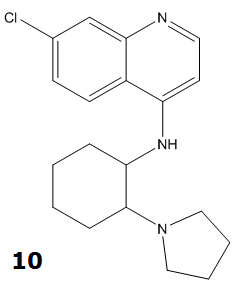 | 7 | 17 | 329.87 |
| 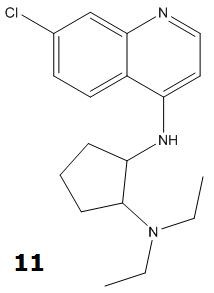 | 5 | 18 | 317.86 |
| 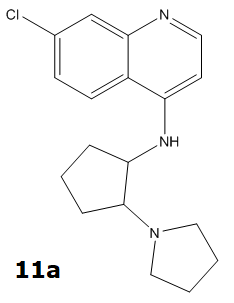 | 7 | 15 | 315.84 |
| 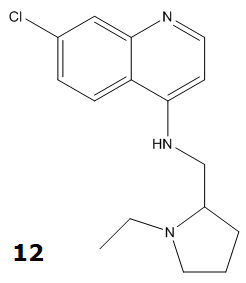 | 6 | 10 | 289.8 |
| 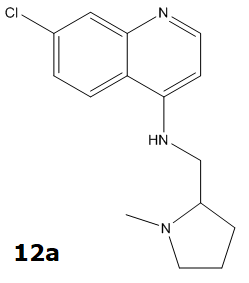 | 7 | 9 | 275.78 |
| 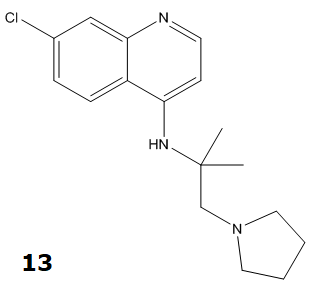 | 30 | 47 | 303.83 |
| 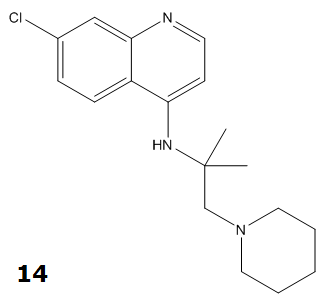 | 9 | 21 | 317.86 |
| 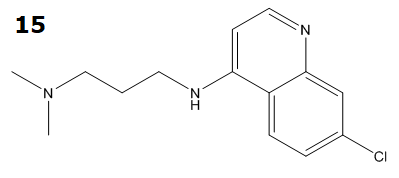 | 2 | 6 | 263.77 |
| 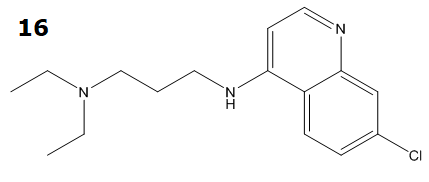 | 3 | 9 | 291.82 |
| 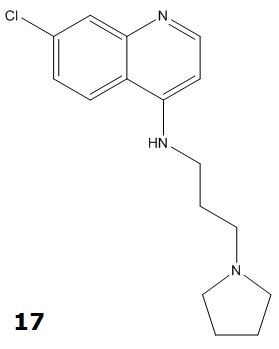 | 3 | 15 | 289.8 |
| 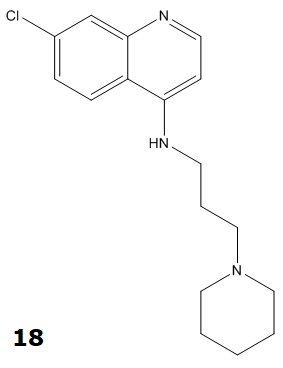 | 4 | 14 | 303.83 |
| 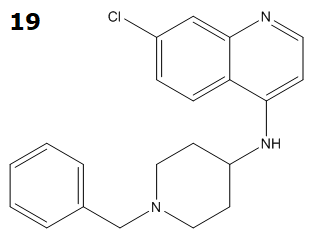 | 7 | 9 | 351.87 |
| 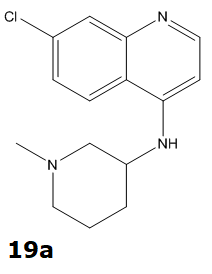 | 6 | 9 | 275.78 |
| 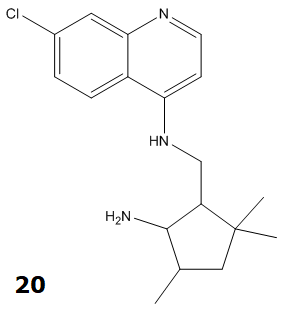 | 3 | 10 | 317.86 |
| 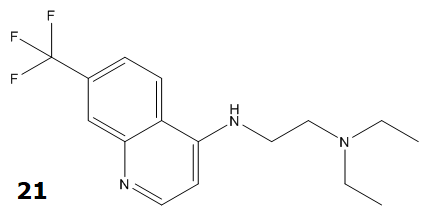 | 21 | 34 | 311.35 |
| 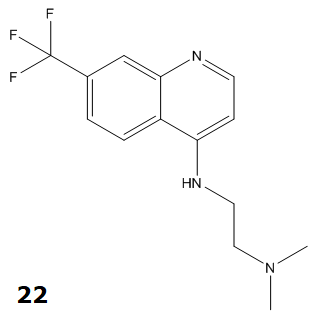 | 14 | 22 | 283.29 |
| 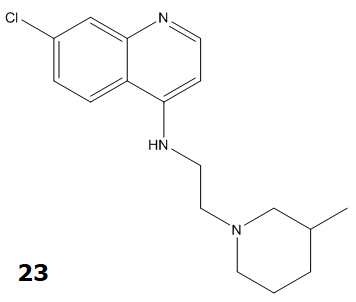 | 6 | 15 | 303.83 |
| 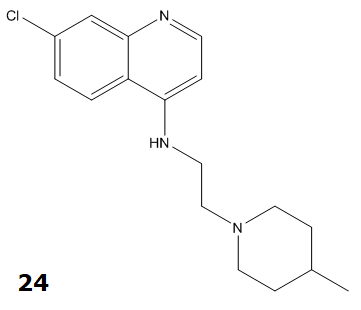 | 7 | 15 | 303.83 |
| 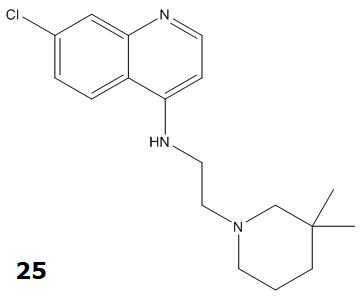 | 8 | 22 | 317.86 |
| 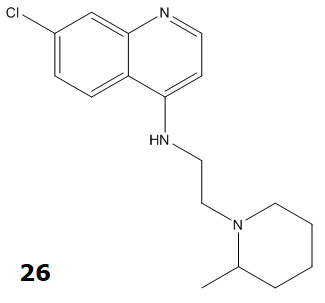 | 7 | 15 | 303.83 |
| 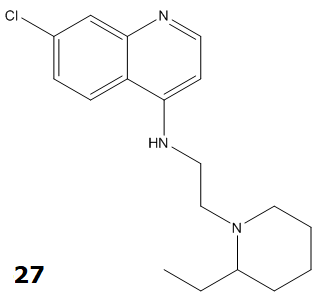 | 8 | 18 | 317.86 |
| 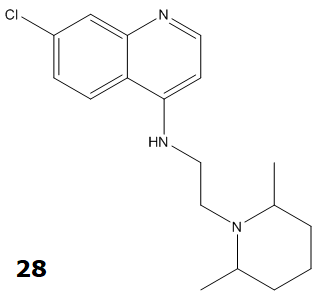 | 7 | 16 | 317.86 |
| 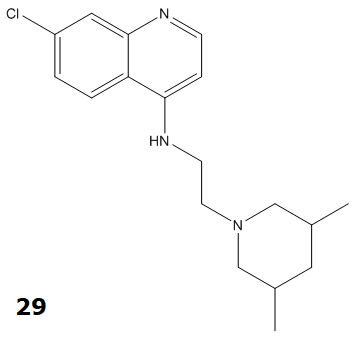 | 6 | 16 | 317.86 |
| 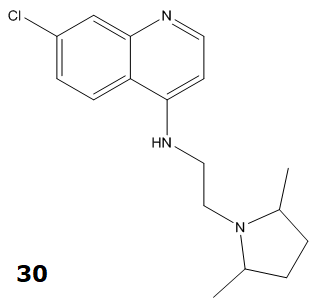 | 7 | 14 | 303.83 |
| 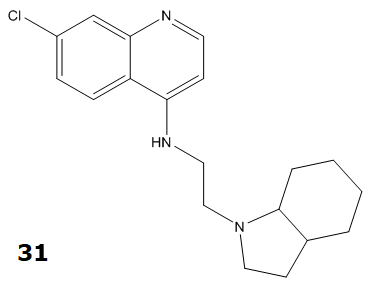 | 5 | 11 | 329.87 |
| 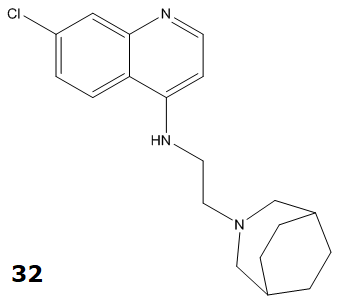 | 7 | 16 | 329.87 |
| 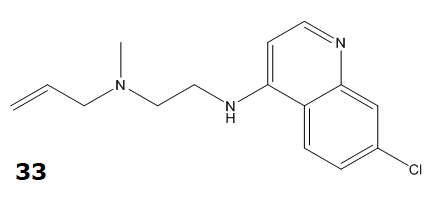 | 8 | 14 | 275.78 |
| 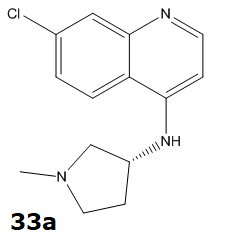 | 7 | 8 | 261.75 |
| 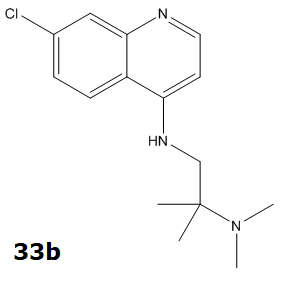 | 8 | 17 | 277.79 |
| 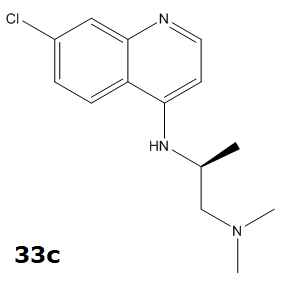 | 8 | 24 | 263.77 |
| 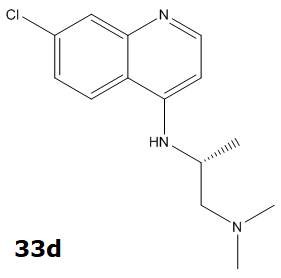 | 8 | 23 | 263.77 |
| 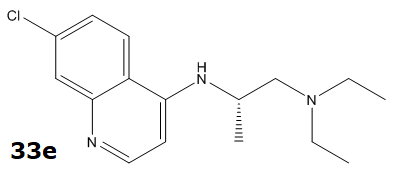 | 12 | 53 | 291.82 |
| 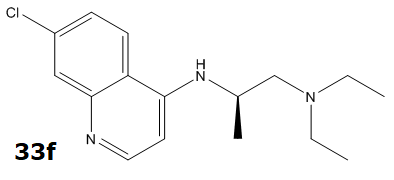 | 12 | 41 | 291.82 |
| 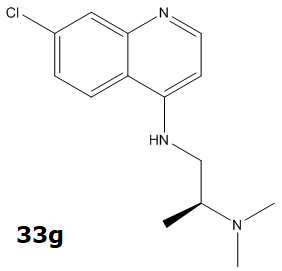 | 5 | 17 | 263.77 |
| 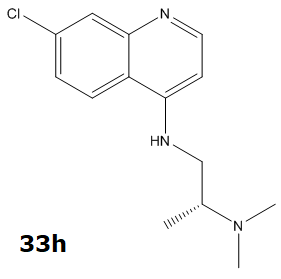 | 7 | 18 | 263.77 |
| 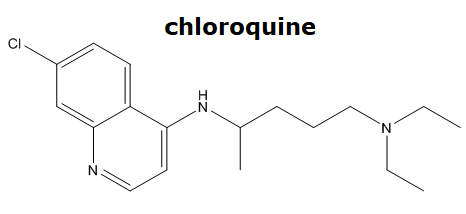 | 8 | 114 | 319.87 |

Table S2 Weights (signed) and standard deviations for the most successful of the partial least squares models for NF54 and K1 pIC50 values. Brief explanations of the descriptors are provided. For further information see Todeschini, R. and Consonni, V., Molecular Descriptors for Chemoinformatics (Wiley-VCH, 2009).

| NF54 (model 19) | (constant: 7.659615) | |  |
| --- | --- | --- | --- |
|  |  |  |  |
| Descriptor | weight (signed) | standard deviation | Brief description |
| Hy | 8.098812×10^−2^ | −8.267599×10^−2^ | Hydrophilic factor |
| Mor31m | −5.441592×10^−2^ | −5.555000×10^−2^ | 3D MoRSE descriptor signal 31 weighted by mass |
| RDF055m | 4.671217×10^−2^ | −4.768570×10^−2^ | Radial Distribution Function weighted by mass |
| JGI5 | −4.627998×10^−2^ | −4.724450×10^−2^ | Mean topological charge index order 5 |
| H-046 | 4.599253×10^−2^ | −4.695107×10^−2^ | H attached to C0(sp3) no X attached next to C |
| GATS7p | 4.405107×10^−2^ | −4.496914×10^−2^ | Geary autocorrelation of lag 7 weighted by polarizability |
| Mor31e | −4.339595×10^−2^ | −4.430037×10^−2^ | 3D MoRSE descriptor weighted by Sanderson electronegativity |
| HARD | −3.973951×10^−2^ | −4.056772×10^−2^ | Hardness |
| GGI1 | −3.684569×10^−2^ | −3.761359×10^−2^ | Topological charge index of order 1 |
| DIPX | −3.651527×10^−2^ | −3.727628×10^−2^ | Dipole Moment X |
| Mor29m | −3.626209×10^−2^ | −3.701782×10^−2^ | 3D-MoRSE descriptor signal 29 weighted by mass |
| H5m | −3.600808×10^−2^ | −3.675852×10^−2^ | GETAWAY descriptor autocorrelation of lag 5 weighted by mass |
| H5p | −3.436139×10^−2^ | −3.507752×10^−2^ | GETAWAY descriptor autocorrelation of lag 5 weighted by polarisability |
| HATS8m | −3.040015×10^−2^ | −3.103372×10^−2^ | GETAWAY descriptor autocorrelation of lag 8 weighted by mass |
| X2Av | 2.797397×10^−2^ | −2.855698×10^−2^ | Average valence connectivity index of order 2 |
| E3u | −2.783525×10^−2^ | −2.841537×10^−2^ | 3rd component accessibility directional WHIM index |
| FDI | −2.760407×10^−2^ | −2.817936×10^−2^ | Distance/distance matrix (G/D) leading eigenvalue from distance/distance matrix |
| DIPY | −2.523651×10^−2^ | −2.576246×10^−2^ | Dipole Moment Y |
| Mor24e | −2.448182×10^−2^ | −2.499205×10^−2^ | 3D-MoRSE descriptor signal 24 weighted by Sanderson electronegativity |
| Rings count | 2.162620×10^−2^ | −2.207691×10^−2^ | Rings count |
| E2s | 2.119065×10^−2^ | −2.163229×10^−2^ | 2nd component accessibility directional WHIM index weighted by I-state |
| H7m | −2.010254×10^−2^ | −2.052150×10^−2^ | GETAWAY descriptor H autocorrelation of lag 7 weighted by mass |
| PCHGMH | −1.993613×10^−2^ | −2.035162×10^−2^ | Mean partial charge on H atoms |
| G3p | 1.668682×10^−2^ | −1.703459×10^−2^ | 3rd component symmetry directional WHIM index weighted by polarizability |
| GATS4e | −1.566494×10^−2^ | −1.599142×10^−2^ | Geary autocorrelation of lag 4 weighted by Sanderson electronegativity |
| C-008 | −1.511917×10^−2^ | −1.543426×10^−2^ | CHR2X |
| SCOUNT(C-atom) | 1.501300×10^−2^ | −1.532589×10^−2^ | Substructure count |
| RDF035e | 1.326425×10^−2^ | −1.354069×10^−2^ | Radial Distribution Function 035 weighted by Sanderson electronegativity |
| RDF050m | 1.225899×10^−2^ | −1.251448×10^−2^ | Radial Distribution Function 050 weighted by mass |
| R1e | 1.221430×10^−2^ | −1.246886×10^−2^ | GETAWAY descriptor R autocorrelation of lag 1 weighted by Sanderson electronegativity |
| Mor20e | 1.159119×10^−2^ | −1.183277×10^−2^ | 3D-MoRSE descriptor signal 20 weighted by Sanderson electronegativity |
| R3u+ | −1.014137×10^−2^ | −1.035272×10^−2^ | GETAWAY descriptor R maximal autocorrelation of lag 3 unweighted |
| G3u | 8.714810×10^−3^ | −8.896436×10^−3^ | WHIM descriptor 3rd component symmetry directional |
| R4p | −8.605457×10^−3^ | −8.784804×10^−3^ | GETAWAY descriptor R autocorrelation of lag 4 weighted by polarizability |
| ENEG | 6.431618×10^−3^ | −6.565659×10^−3^ | Electronegativity |
| DIP | 5.434864×10^−3^ | −5.548133×10^−3^ | Dipole Moment |
| HATS7m | 4.885984×10^−4^ | −4.987813×10^−4^ | GETAWAY descriptor leverage-weighted autocorrelation of lag 7 weighted by mass |
| SCOUNT(N-atom) | 0 | 0 | Substructure count |

Table S2 (continued)

| K1 (model 20) |  | | (constant: 7.278025) | |
| --- | --- | --- | --- | --- |
|  |  |  | |  |
| Descriptor | weight (signed) | standard deviation | | Brief description |
| Mor31e | −8.279356×10^−2^ | −7.520413×10^−2^ | | 3D MoRSE descriptor weighted by Sanderson electronegativity |
| HARD | −8.246118×10^−2^ | −7.490222×10^−2^ | | Hardness |
| Mor31m | −6.313831×10^−2^ | −5.735062×10^−2^ | | 3D MoRSE descriptor signal 31 weighted by mass |
| Count of rotatable bond | −5.962762×10^−2^ | −5.416174×10^−2^ | | Count of rotatable bond |
| RDF030m | 5.608514×10^−2^ | −5.094399×10^−2^ | | Radial Distribution Function 030 weighted by mass |
| BELp3 | 5.600810×10^−2^ | −5.087401×10^−2^ | | Lowest eigenvalue n. 3 of Burden matrix weighted by atomic polarizabilities |
| J3D | −5.427589×10^−2^ | −4.930059×10^−2^ | | Balaban-like index from geometrical matrix 3D matrix-based descriptors |
| HATS7p | −4.883058×10^−2^ | −4.435443×10^−2^ | | GETAWAY descriptor leverage-weighted autocorrelation of lag 7 weighted by polarisability |
| R8e | −4.511822×10^−2^ | −4.098237×10^−2^ | | GETAWAY descriptor R autocorrelation of lag 8 weighted by Sanderson electronegativity |
| H5p | −4.391553×10^−2^ | −3.988993×10^−2^ | | GETAWAY descriptor H autocorrelation of lag 5 weighted by polarizability |
| Rings count | 4.351619×10^−2^ | −3.952719×10^−2^ | | Rings count |
| DIPY | −3.558066×10^−2^ | −3.231909×10^−2^ | | Dipole moment Y |
| LOGP | −3.392758×10^−2^ | −3.081755×10^−2^ | | Moriguchi octanol-water partition coefficient |
| Mor04e | 3.334596×10^−2^ | −3.028924×10^−2^ | | 3D MoRSE descriptor signal 4 weighted by Sanderson electronegativity |
| nCt | −3.264431×10^−2^ | −2.965191×10^−2^ | | Number of total tertiary C(sp3) |
| As | −3.198719×10^−2^ | −2.905502×10^−2^ | | WHIM descriptor A total size weighted by I-state |
| E3u | −3.080757×10^−2^ | −2.798353×10^−2^ | | WHIM descriptor 3rd component accessibility directional |
| GGI1 | −2.943023×10^−2^ | −2.673245×10^−2^ | | Topological charge autocorrelations index of order 1 |
| ENEG | 2.673222×10^−2^ | −2.428176×10^−2^ | | Electronegativity |
| R7u+ | −2.447716×10^−2^ | −2.223341×10^−2^ | | GETAWAY descriptor R maximal autocorrelation of lag 7 unweighted |
| DIPX | −2.397050×10^−2^ | −2.177320×10^−2^ | | Dipole moment X |
| Mor20e | 2.249459×10^−2^ | −2.043258×10^−2^ | | 3D MoRSE descriptor signal 20 weighted by Sanderson electronegativity |
| Mor11e | −2.069688×10^−2^ | −1.879966×10^−2^ | | 3D MoRSE descriptor signal 11 weighted by Sanderson electronegativity |
| PCHGPH | −1.972265×10^−2^ | −1.791474×10^−2^ | | Mean partial charge on H atoms |
| PW2 | 1.889137×10^−2^ | −1.715966×10^−2^ | | Path/walk indices path/walk 2 Randic shape index |
| RTu+ | 1.855476×10^−2^ | −1.685390×10^−2^ | | GETAWAY descriptor R maximal autocorrelation unweighted |
| R2u | 1.567099×10^−2^ | −1.423448×10^−2^ | | GETAWAY descriptor R autocorrelation of lag 2 unweighted |
| G2v | 1.563596×10^−2^ | −1.420266×10^−2^ | | WHIM descriptor 2nd component symmetry directional weighted by van der Waals volume |
| FDI | −1.196298×10^−2^ | −1.086637×10^−2^ | | Distance/distance matrix (G/D) leading eigenvalue from distance/distance matrix |
| R8u+ | −9.157233×10^−3^ | −8.317818×10^−3^ | | GETAWAY descriptor R autocorrelation of lag 8 unweighted |
| RDF065m | 6.460672×10^−3^ | −5.868443×10^−3^ | | Radial Distribution Function 065 weighted by mass |
| SCOUNT(C-atom) | −2.090814×10^−3^ | −1.899156×10^−3^ | | Substructure count |
| PCHGMHT | 9.051149×10^−4^ | −8.221458×10^−4^ | | Mean partial charge on heteroatoms |
